# Reproducibility of human cardiac phosphorus MRS (^31^P‐MRS) at 7 T

**DOI:** 10.1002/nbm.4095

**Published:** 2019-03-29

**Authors:** Jane Ellis, Ladislav Valkovič, Lucian A.B. Purvis, William T. Clarke, Christopher T. Rodgers

**Affiliations:** ^1^ OCMR, RDM Cardiovascular Medicine University of Oxford UK; ^2^ Slovak Academy of Sciences Institute of Measurement Science Bratislava Slovakia; ^3^ Wellcome Centre for Integrative Neuroimaging, FMRIB, Nuffield Department of Clinical Neurosciences University of Oxford UK; ^4^ Wolfson Brain Imaging Centre, Department of Clinical Neurosciences University of Cambridge UK

**Keywords:** ^31^P, 7 T, cardiac, human, MRS, reproducibility

## Abstract

**Purpose:**

We test the reproducibility of human cardiac phosphorus MRS (^31^P‐MRS) at ultra‐high field strength (7 T) for the first time. The primary motivation of this work was to assess the reproducibility of a ‘rapid’ 6½ min ^31^P three‐dimensional chemical shift imaging (3D‐CSI) sequence, which if sufficiently reproducible would allow the study of stress‐response processes. We compare this with an established 28 min protocol, designed to record high‐quality spectra in a clinically feasible scan time. Finally, we use this opportunity to compare the effect of per‐subject *B*_0_ shimming on data quality and reproducibility in the 6½ min protocol.

**Methods:**

10 healthy subjects were scanned on two occasions: one to test the 28 min 3D‐CSI protocol, and one to test the 6½ min protocol. Spectra were fitted using the OXSA MATLAB toolbox. The phosphocreatine to adenosine triphosphate concentration ratio (PCr/ATP) from each scan was analysed for intra‐ and intersubject variability. The impact of different strategies for voxel selection was assessed.

**Results:**

There were no significant differences between repeated measurements in the same subject. For the 28 min protocol, PCr/ATP in the midseptal voxel across all scans was 1.91 ± 0.36 (mean ± intersubject SD). For the 6½ min protocol, PCr/ATP in the midseptal voxel was 1.76 ± 0.40. The coefficients of reproducibility (CRs) were 0.49 (28 min) and 0.67 (6½ min). Per‐subject *B*_0_ shimming improved the fitted PCr/ATP precision (for 6½ min scans), but had negligible effect on the CR (0.67 versus 0.66).

**Conclusions:**

Both 7 T protocols show improved reproducibility compared with a previous 3 T study by Tyler et al. Our results will enable informed power calculations and protocol selection for future clinical research studies.

**Abbreviations used**
2,3‐DPG: 2,3‐diphosphoglycerate
^31^P‐MRS: phosphorus MRS
3D‐CSI: three‐dimensional chemical shift imaging
ATP: adenosine triphosphate
CR: coefficient of reproducibility
CRLB: Cramér‐Rao lower bound
CSI: chemical shift imaging
CV: coefficient of variation
DCM: dilated cardiomyopathy
FLASH: fast low‐angle shot
GRE: gradient‐recalled echo
OXSA: Oxford Spectroscopy Analysis
PCr: phosphocreatine
PPA: phenylphosphonic acid
SD: standard deviation
SNR: signal‐to‐noise ratio
UTE‐CSI: ultra‐short echo chemical shift imaging
WSVD: whitened singular value decomposition.

## 1 INTRODUCTION

Phosphorus MRS (^31^P‐MRS) is a non‐invasive technique used to measure the concentrations and chemical kinetics of high‐energy phosphorus‐containing metabolites in the human heart, often collectively referred to as ‘cardiac energetics’. ^31^P‐MRS has provided a unique insight into our understanding of cardiac metabolism.^1^, ^2^ The application of ^31^P‐MRS to cardiovascular research is of interest, since in most major heart diseases the ratio of phosphocreatine (PCr) to adenosine triphosphate (ATP) concentrations (PCr/ATP) changes, making it a useful indicator of the altered energetic state of the heart. Examples of diseases where PCr/ATP decreases include Type I and Type II diabetes,^3^, ^4^ hypertensive heart disease,^5^ coronary artery disease^6^, ^7^ and heart failure.^8^, ^9^

However, ^31^P‐MRS is yet to be translated into routine use in a clinical setting, owing mainly to its intrinsically low signal‐to‐noise ratio (SNR). According to theory, the quality of the raw ^31^P‐MRS signal ( $\mathrm{SNR}∕\sqrt{T_{A}}$, where *T*_A_ is scan duration) increases approximately linearly with the scanner's magnetic field strength *B*_0_.^10^ Accordingly, ^31^P‐MRS benefits significantly from a move to ultra‐high field strengths, ie *B*_0_ ≥ 7 T. At 7 T, an increase in SNR of 2.8‐fold in the human heart compared with 3 T has recently been demonstrated,^11^ allowing the acquisition in 6 min of spectra of a quality comparable to that of spectra that took 31 min at 3 T. Acquiring usable ^31^P spectra in shorter scan times is highly desirable; a finer temporal resolution would allow study of the response cardiac energetics to stressors (eg dobutamine infusion or exercise).

It is, however, questionable whether such a short protocol would be sufficiently robust and reproducible to detect changes in cardiac metabolism with sufficient power. Therefore, the primary motivation of this work was to assess reproducibility values of PCr/ATP ^31^P‐MRS measurements at 7 T in the human heart for a ‘rapid’ 6½ min three‐dimensional chemical shift imaging (3D‐CSI) sequence. If found to be sufficiently reproducible, this would allow the study of multiple steady states within a single scanning session. We compare this with an established 28 min protocol that was designed to record high‐quality spectra in a scan time that is tolerable to a majority of patients.^11^ This will allow informed decisions when selecting protocols in the design of future studies. We also evaluate the effect of per‐subject *B*_0_ shimming on spectral quality and reproducibility. Finally, we illustrate the impact of these technical improvements by comparing the sample sizes and approximate scan costs of studies using these optimized 3 T and 7 T protocols.

## 2 EXPERIMENT

**Figure 1:**
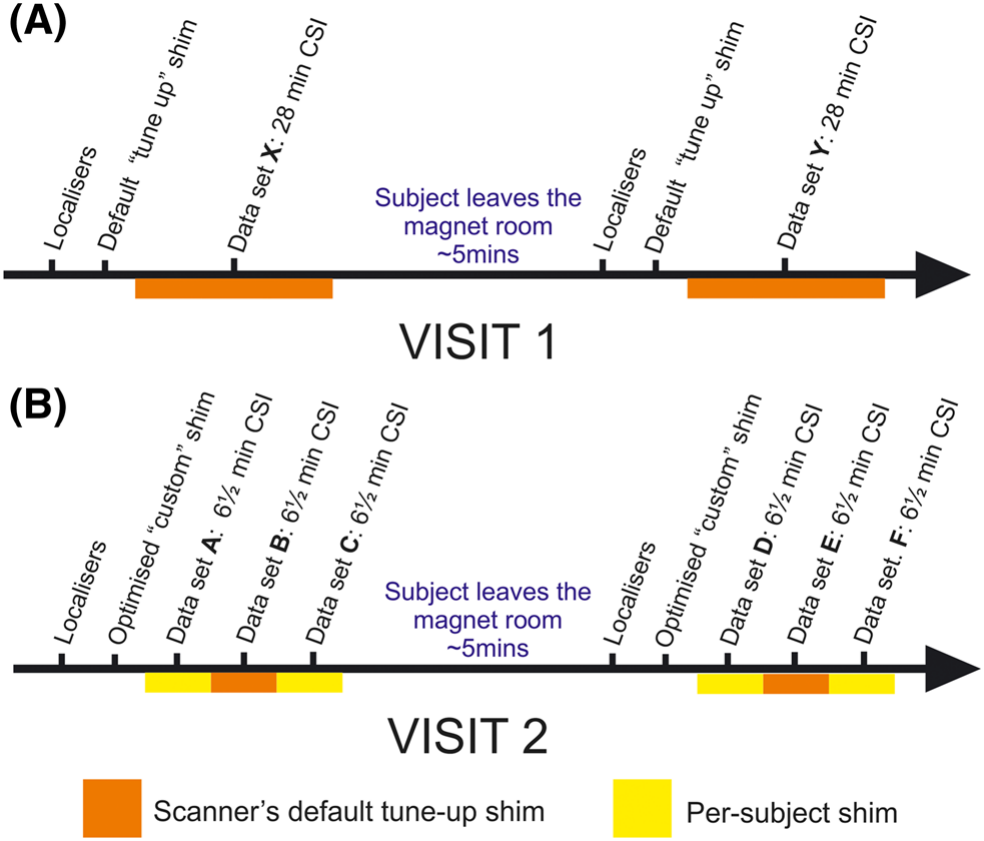
Study protocol. A ‘session’ was defined as a series of consecutive sequences, acquired without the patient leaving the magnet. ‘Dataset’ refers to a single CSI sequence acquired within a session. A, the visit protocol for acquisition of 28 min CSI datasets; B, the 6½ min datasets

Ten healthy volunteers (three female, age 29 ± 6 years, BMI 23 ± 4 kg/m^2^) were recruited according to local ethics regulations. Volunteers made two separate visits. On each visit they underwent two scanning sessions as shown in Figure 1. On the first visit, two 28 min ^31^P‐MRS spectra were acquired (one during each scan session) using the manufacturer's default shim settings. On the second visit, three 6½ min ^31^P‐MRS spectra were acquired in both scanning sessions. During the second visit, we also tested a customized *B*_0_ shimming algorithm against the manufacturer's default shim settings.

### 2.1 Data acquisition

All scans were performed on a Magnetom 7 T MRI scanner (Siemens, Erlangen, Germany). To facilitate coil swapping, subjects were scanned head‐first supine. A 10 cm ^1^H transmit/receive loop coil (Rapid Biomedical, Rimpar, Germany) was used to acquire two‐chamber, four‐chamber and mid‐short‐axis ^1^H fast low‐angle shot (FLASH) stacks of localizer cine images.

*B*_0_ maps were acquired as a stack of 18 dual‐echo gradient‐recalled echo (GRE) slices aligned with the mid‐short‐axis view (*T*_R_ 314 ms, *T*_E1_ 2.4 ms, *T*_E2_ 4.3 ms). *B*_0_ maps were measured starting from the scanner manufacturer's default shim settings (the ‘tune‐up’ shim settings). Per‐subject shim solutions were obtained by recording a *B*_0_ map using a dual‐echo GRE shim work‐in‐progress package (WIP 452B ‘GRE SHIM’, Siemens). This field map was altered in MATLAB (MathWorks, Natick, MA, USA) by zeroing all pixels with an intensity less than that in the inferior myocardium and setting the magnitude of all remaining pixels to 1. The phases of the pixels were left unaltered. This modified *B*_0_ map was then used as input to the scanner's standard shim calculation routines. These modifications prevent the very high‐intensity pixels near the surface dominating the computed shim solution, which might otherwise degrade the shim quality in the inferior cardiac segments.

The ^1^H coil was replaced with a 16‐element ^31^P receive array coil (Rapid Biomedical, Rimpar, Germany), consisting of a single rectangular 28 × 27 cm^2^ transmit loop and a 4 × 4 matrix of 16 circular flexible receive loops 5.5 cm in diameter. The coil was placed in the same position above the mid‐ventricular septum.^12^ The transmit efficiency was calibrated per‐subject by using a series of inversion‐recovery free induction decays to acquire signal from a central spherical phenylphosphonic acid (PPA) fiducial mounted on the coil housing and processed with custom MATLAB code. The coil position was determined from three orthogonal single‐channel ^31^P FLASH images, which localize five PPA fiducials (including the centre fiducial used to compute the *B*_1_ calibration) using custom MATLAB code.

A 25 mm thick, *B*_1_‐insensitive train to obliterate signal (BISTRO) saturation band was placed in the anterior chest wall to suppress signal from skeletal muscle as previously described.^13^ The voltage of the saturation pulse was set to the maximum value allowed to comply with the legal specific absorption rate limits in each subject. Excitation was placed at +266 Hz relative to PCr so as to cover metabolites from 2,3‐diphosphoglycerate (2,3‐DPG) to γ‐ATP. The CSI grid was positioned in the short‐axis view of the heart, such that the longest voxel dimension was aligned with the intraventricular septum and the in‐plane voxel matrix was parallel to the chest wall. The CSI matrix was fixed at the point of acquisition, and not shifted in post‐processing. Respiratory gating and ECG triggering were not used.

In the first visit, a single ^31^P dataset was acquired in 28 min with a 3D ultra‐short echo (UTE)‐CSI sequence with the following parameters: matrix size 16 × 16 × 8; nominal voxel size 15 × 15 × 25 mm^3^; acquisition weighting with 10 averages at *k* = 0; repetition time 1 s and whitened singular value decomposition (WSVD) coil combination.^14^ The excitation was set to 400 V peak amplitude (ie 3.2 kW), giving a flip angle of approximately 30^0^ in the interventricular septum. RF excitation was performed using a shaped pulse that has been previously described.^15^ It comprises a 0.5 ms hard pulse, preceded by a numerically optimized 1.9 ms part that improves homogeneity of excitation. It excites an approximately 2 kHz bandwidth. Subjects were then removed from the magnet—this constituted Session 1. After a short (~5 min) break, localization was repeated and an identical 28 min 3D UTE‐CSI sequence was run in Session 2. These datasets are labelled X and Y (see Figure 1 for detailed description).

During the second visit, three sets of ^31^P spectra were acquired in 6½ min each. The same 3D UTE‐CSI sequence was used as described above, but with an 8 × 16 × 8 matrix; nominal voxel size 25 × 15 × 25 mm^3^ and four averages (*k* = 0) to enable the shorter acquisition time. Using larger voxels allowed us to reduce scan duration while keeping enough samples at *k* = 0 to preserve a compact voxel point‐spread‐function with minimal side‐lobes. The first and third datasets used per‐subject *B*_0_ shimming; the second used the vendor's standard tune‐up shim settings. As in the first visit, subjects were then removed from the magnet—this constituted Session 3. After a short (~5 min) break, these steps were repeated as Session 4. These datasets are labelled A‐F (see Figure 1 for detailed description).

### 2.2 Data analysis

Four voxels in the mid‐interventricular septum were identified for further analysis: the midseptal, anteroseptal, anterior and posterior voxels. These were assigned anatomically; the midseptal voxel was two voxels posterior to the chest wall. Data from these voxels were fitted using the Oxford Spectroscopy Analysis (OXSA) toolbox's implementation of AMARES.^16^, ^17^ Prior knowledge specified 11 Lorentzian peaks, fixed amplitude ratios, and literature values for the scalar couplings for the multiplets. Blood contamination and partial saturation were corrected using *T*_1_ values from the literature.^11^, ^15^ All scans were included in the analysis. PCr/ATP is reported as the blood‐ and saturation‐corrected values of PCr/γ‐ATP, excluding the α‐ATP peak because it has contributions from NADH, and the β‐ATP peak because it was outside the uniform flip‐angle bandwidth of the excitation pulse at 7 T. Cramér‐Rao lower bounds (CRLBs) were used to express the uncertainty in metabolite concentrations.^18^ Reproducibility statistics of the four identified voxels and their spectral sum were calculated.

### 2.3 Assessment of reproducibility

*Intersession variability* was assessed through the mean and difference between PCr/ATP ratios from equivalent datasets in both protocols for each subject, ie by comparing Dataset B with E, C with F, and X with Y (see Figure 1 for detailed description).

*Intersubject variability* was assessed by the mean and standard deviation (SD) of PCr/ATP ratios within the same datasets across all subjects.

*Intrasession variability*, only applicable to the second visit, was assessed through the mean and difference between PCr/ATP ratios from equivalent datasets within the same session, ie by comparison of Dataset A with C and Sataset D with F.

The coefficient of reproducibility (CR) was calculated from SD of the *signed* differences in PCr/ATP between two scans for each subject according to

(1) $$\mathrm{CR}={\mathrm{SD}}_{\text{intrasubject}}\times 1.96.$$

A *lower* CR reflects a better method. A two‐tailed Mann–Whitney *U* (non‐parametric) test was used to compare repeated measurements. Variances were compared using a Brown‐Forsythe test.^19^ The coefficient of variation (CV), defined as the sample SD divided by the mean, is also reported.

(2) CV=SD/mean.

Datasets X and Y were compared with those collected from 25 patients with dilated cardiomyopathy (DCM) in a previous study using the same 28 min protocol, coils and scanner.^20^

## 3 RESULTS

**Figure 2:**
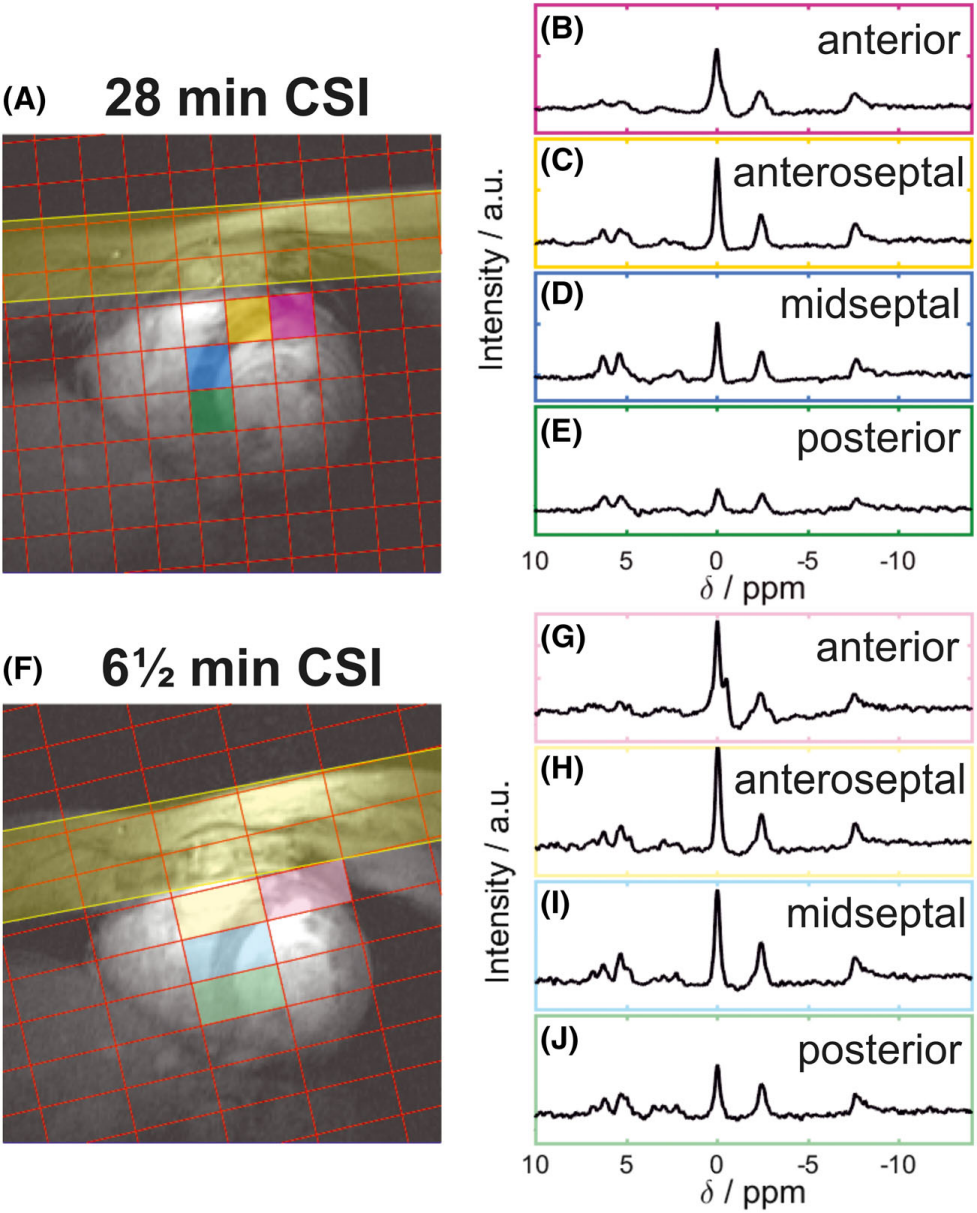
A, F, positions of the CSI matrix showing the rotation of the CSI grid in the short‐axis view of the heart for both protocols. B‐E, G‐J, spectra from the corresponding coloured voxel marked on the localizer images

Typical spectra from the 28 min and the 6½ min CSI protocols (using the tune‐up shim) from across the heart shown are shown in Figure 2. Lower signal is observed in the posterior voxels owing to the use of a surface coil. Across all subjects, the SNR of PCr was 1.2 times greater in the 28 min CSI scan compared with the 6½ min scan (having fewer averages but larger voxels). Furthermore, in the 6½ min scan we observed a larger 2,3‐DPG amplitude (3.5 ± 0.62 versus 2.9 ± 1.06, *P* = 0.07), reflecting greater blood contamination from the larger voxels.

### 3.1 Analysis methods

**Figure 3:**
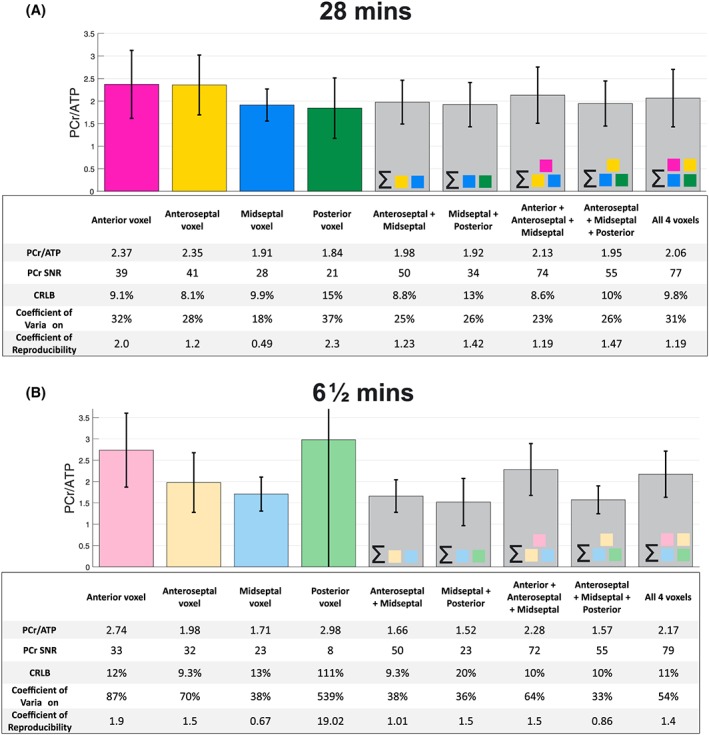
Variation in PCr/ATP value measured at four voxel locations and combinations of spectral sums of these voxels for 28 min CSI (A) and 6½ min CSI (B). The tables below provide the PCr SNR, the CRLB on PCr, the CV (intersubject SD/mean) and the CR (1.96 × interscan SD) of the measured PCr/ATP. Please note that the values here refer to interexamination repeatability

Figure 3 gives reproducibility statistics for single voxels and for combinations of voxels summed prior to fitting. For both protocols, the midseptal voxel gives the most reproducible PCr/ATP values, shown by the lowest CR. The voxels closest to the chest wall (the anterior and anteroseptal voxels) have the highest PCr SNR, and the lowest PCr/ATP CRLB, indicating a more precise quantification. However, the reproducibility is lower in these voxels than in the midseptal voxel. The posterior voxel has both a lower quantification precision (higher CRLBs) and worse reproducibility than the mid‐septal voxel. Summing spectra from the midseptal voxel with anteroseptal and anterior voxels before fitting leads to a reduction in PCr/ATP CRLB but comes at a cost to the reproducibility of the measurement. All subsequent analyses therefore calculated PCr/ATP from the midseptal voxel, not the summed voxels.

### 3.2 Assessment of reproducibility

#### 3.2.1 28 min protocol

**Figure 4:**
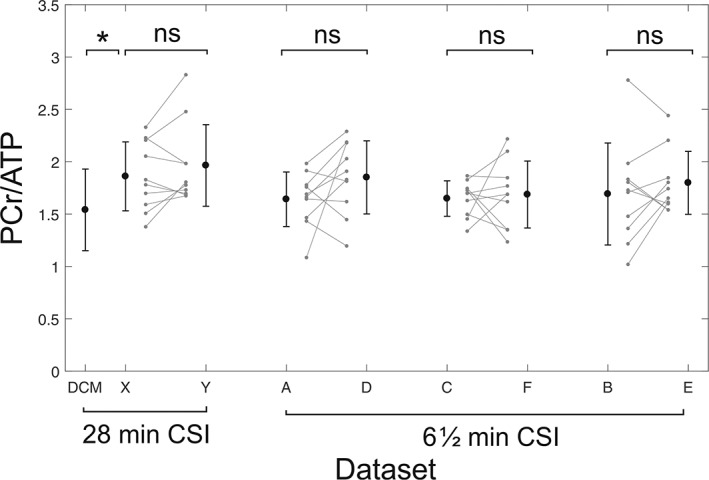
Intersubject variability between scans for the 28 min CSI protocol (left) and the 6½ min CSI protocol (right). Error bars show ±1 SD. The leftmost bar shows DCM patient data acquired by Stoll et al^20^ using the same scanning protocol and hardware for comparison

**Figure 5:**
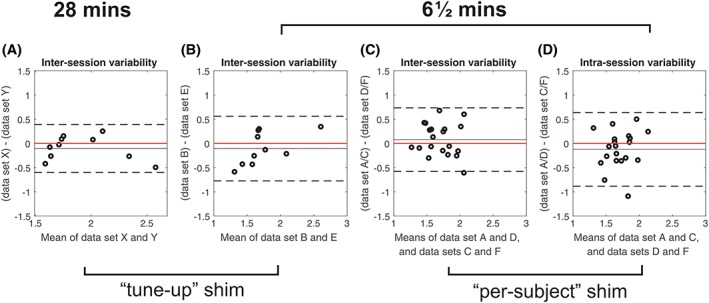
A‐C, bland–Altman plots of intersession variability in PCr/ATP for 28 min CSI (a) and 6½ min CSI (B, C). D, plot of intrasession variability for 6½ min CSI with per‐subject shimming. Solid black lines show the bias from zero (red line); dashed lines mark ±1.96 SD

There were no significant differences in PCr/ATP (1.86 ± 0.32 versus 1.96 ± 0.39, *P* = 0.79) between repeated measurements in the same subjects (Figure 4). The mean PCr/ATP measured across all scans was 1.91 ± 0.36, giving a CV of 18%. The intrasubject variability was 0.21 ± 0.16, giving a CR of 0.49. These values are shown on Bland–Altman plots in Figure 5.

#### 3.2.2 6½ min protocol

There were no significant differences (Figure 4) between repeated measurements using the default tune‐up shim (1.69 ± 0.48 versus 1.79 ± 0.30, *P* = 0.57) or the customized shimming (1.64 ± 0.26 versus 1.65 ± 0.17, *P* = 0.97). Mean PCr/ATP across all scans was 1.76 ± 0.40 for the tune‐up and 1.70 ± 0.28 for the custom shim, giving CVs of 23% and 17% respectively. The intrasubject variabilities were 0.31 ± 0.14 (tune‐up) and 0.29 ± 0.18 (per‐subject shim). There was an improvement in the CRLBs between tune‐up and the customized shimming algorithms (13.8% versus 10.3%, *P* = 0.02), showing that the higher‐quality spectra obtained using the customized shimming enabled more precise metabolite quantification. The reproducibilities of PCr/ATP measurements using the two shimming techniques were equivalent (CR 0.66 tune‐up shim versus 0.67 custom shim).

The interexamination CR was 0.75 and the intraexamination CR was 0.67 (both reported for the custom shim comparison). There was no significant difference (*P* = 0.85) between these inter‐ and intraexamination changes.

### 3.3 Power calculations and sample size

Sample sizes providing sufficient power to reveal differences in PCr/ATP for paired (eg response to stressors or patient longitudinal cohort) studies are given in Table 1. These were calculated using the reproducibility values obtained in this work and literature results at 3 T.

**Table 1:** Power calculations showing the sample size needed in a paired study to determine statistical significance (α = 0.05) for both 28 min and 6.5 min CSI sequences at 7 T, and 31 min CSI sequence at 3 T, for a change of 0.2 in the measured PCr/ATP

**ΔPCr/ATP = 0.2**	**7 T**		**3 T** a
	**28 min σ = 0.25, α = 0.05**	**6½ min σ = 0.34, α = 0.05**	**31 min σ = 0.56, α = 0.05**
Power 95%	23	40	104
Power 90%	19	33	85
Power 80%	15	25	64

a 3 T reproducibility values for this power calculation were taken from the study by Tyler et al.^15^ We made this analysis using the original raw data from that study provided by Professor Tyler. If comparing this table with Reference 15, please note that the values reported in Table 1 of Reference 15 were computed using the SD of the absolute difference in PCR/ATP for Scan 1 and Scan 2, whereas here we have used the SD of the signed difference.

## 4 DISCUSSION

We have tested the reproducibility of a ‘rapid’ 6½ min 3D‐CSI protocol, which is short enough to allow the assessment of changes in PCr/ATP during rest, stress, and recovery in a single scanning session. We also tested reproducibility for an established 28 min 3D‐CSI protocol that was designed to give the best spectra in an examination tolerable to cardiac patients. We believe that this study reports the first reproducibility data for human cardiac ^31^P‐MRS at 7 T. These are crucial for planning future clinical studies.

Reproducibility of human cardiac ^31^P‐MRS has previously been reported at lower field strengths (summarized in Table 2). These studies encompass a range of field strengths, localization methods and voxel volumes, which makes it hard to draw quantitative conclusions when comparing these studies. However, it is clear that our 28 min protocol has the tightest reproducibility of all the 3D‐resolved protocols, and that it achieves this with a small 5.6 mL nominal voxel volume.

**Table 2:** Reproducibility values of human cardiac ^31^P‐MRS from the literature

**Reference**	**Field strength (T)**	**Cohort size**	**Acquisition time (min)**	**Localization method**	**Nominal voxel size (cm** ^**3**^ **)**	**Absolute reproducibility (%)** a
This study	7	10	28	3D‐CSI	1.5 × 1.5 × 2.5 = 5.6 mL	26
			6½	3D‐CSI	2.5 × 1.5 × 2.5 = 9.4 mL	39
Bakermans et al, 2017^24^	3	8	7	Single‐voxel 3D ISIS	(8.0)^3^ = 512 mL	40
				1D ISIS with 1D CSI	—	43
				1D ISIS with 1D CSI	—	44
Tyler et al, 2009^15^	3	20	30	3D CSI	1.5 × 1.5 × 2.5 = 5.6 mL	53
Abozguia et al, 2010^29^	3	1^b^	23	3D ISIS	4.4 × 5.5 × 3.7 = 90 mL	31
Schaefer et al, 1992^30^	1.5	7	7	1D CSI	—	22
Lamb et al, 1996^25^	1.5	16	10	3D ISIS	6.0 × 7.0 × 7.0 = 294 mL	38^c^
			15	1D CSI	—	—
			30	2D ISIS +1D CSI	1.0 cm thick slices	46^c^

a Absolute reproducibility is calculated as [CR/(mean PCr/ATP)] × 100%.

b A single subject was scanned eight times to assess reproducibility.

c Absolute reproducibility was computed by extracting raw values from Figure 5 in Reference 25 using WebPlotDigitizer (https://apps.automeris.io/wpd/).

### 4.1 Analysis methods

The midseptal voxel is the most reproducible of all the single voxels and gives PCr/ATP consistent with literature values. The anterior and anteroseptal voxels have higher PCr/ATP values and we hypothesize that this is due to contamination of the ‘cardiac’ spectra by small amounts of skeletal muscle signal that may not be fully suppressed in some scans by the saturation bands (PCr/ATP is approximately 4–5 in skeletal muscle versus 2 in myocardium).^21^ Quantification of PCr/ATP values in the posterior voxels is the least reproducible, and for the 6½ min protocol has an extremely large CRLB (111%). This is probably due to a combination of the effects of distance from the surface coil, and of motion in the posterior voxel.

In 2009, Tyler et al performed a cardiac ^31^P‐MRS reproducibility study at 3 T.^15^ They used the spectral sum from three voxels in their analysis because they observed lower PCr/ATP CRLB than for a single midseptal voxel. They attributed this increase in precision to the gain in SNR from combining signals. In this study, at 7 T, we also saw increased SNR on summing voxels, but no corresponding improvement in reproducibility.

Our analysis method introduces minimal user bias: no spectra were excluded from the study based on appearance, and as the rest of the fitting in OXSA is automated the measured PCr/ATP is only dependent on which voxel was selected for analysis. However, as measured PCr/ATP varied at differing anatomical locations across the heart, the selection of the voxel is an important step. It was therefore important to define anatomically which voxel would be selected for analysis at the start of the study, and ensure that it was used in all subjects.

### 4.2 Assessment of reproducibility

#### 4.2.1 28 min protocol

Measurements reported here are consistent with literature values.^11^ While repeated measurements of PCr/ATP measured here showed no significant differences from each other, PCr/ATP measured in this work did show significant difference from values measured in 25 DCM patients using an identical 28 min CSI protocol and the same hardware (1.91 ± 0.36 versus 1.54 ± 0.39, *P* = 0.01).^9^

Despite SNR limitations, 3 T ^31^P‐MRS has allowed observation of cardiac energetic changes in many disease groups. In recent years, 3 T has been the most widely used field strength for cardiac phosphorus scans. The 28 min CSI protocol tested here has equal voxel sizes (5.6 mL nominal) and a similar scan duration (28 versus 31 min) to the 3 T protocol tested by Tyler et al.^15^ In that study, they found intrasubject percentage differences of 20% (absolute difference 0.43, mean PCr/ATP 2.10) and a CR of 1.1. In this study, we report lower intrasubject percentage differences of 11% (absolute difference 0.21, mean PCr/ATP 1.91) and a CR of 0.49 (ie more reproducible).

#### 4.2.2 6½ min protocol

The ‘rapid’ 6½ min CSI acquired datasets in less than one‐quarter of the time of both those in the 3 T study by Tyler et al and the 28 min scan tested here. As expected, a decrease in scan time led to increased variability in PCr/ATP measurements and therefore a larger CR (0.49 for 28 min CSI versus 0.67 for 6½ min CSI). Despite this, the reproducibility of PCr/ATP measurements acquired in 6½ min at 7 T is still greater than that of measurements acquired in 31 min at 3 T, so a move to ultra‐high field means that data can be acquired more quickly without cost to reproducibility. This is important as short ^31^P acquisition times allow the detailed study of the response of cardiac energetics to stressors, such as exercise or dobutamine infusion. For example, the British Society of Echocardiography's recommendations for a dobutamine protocol for assessment of myocardial ischemia are to use four steps each with 3 min of dobutamine infusion.^22^ 7 T ^31^P stress using our 6½ min ^31^P scan would therefore be possible during dobutamine stress complying with these guidelines, whereas a 31 min scan at 3 T would not be. We note in passing that Dass et al proposed a shorter 8 min ^31^P scan at 3 T, and demonstrated that it gave similar mean PCr/ATP values to the Tyler et al 31 min 3 T protocol (Dass et al 8 min, 1.83 ± 0.32; Tyler et al 31 min, 1.78 ± 0.27).^23^ However, the reproducibility of that 8 min 3 T protocol has not been reported, so we cannot compare its reproducibility against our 7 T results.

Bakermans et al tested the reproducibility of a 7 min sequence in the human heart at 3 T, performing spatial localization with 3D ISIS or a combination of 1D ISIS and 1D CSI either perpendicular or parallel to the surface coil.^24^ 3D ISIS was found to be the most reproducible of these methods, giving a PCr/ATP of 1.57 ± 0.17 (mean ± SD) and a CR of 0.64. The reproducibility of this 7 min 3 T scan is the same as that achieved in a 6½ min 7 T scan in this work (CR 0.64 at 3 T versus CR 0.67 in this work) but used substantially larger voxel sizes: 3D ISIS voxel size 512 mL.

We observed no significant differences between the variances of the intra‐ and inter examination differences (*P* = 0.85), suggesting that the variability in measured PCr/ATP is dominated by error from within the ^31^P measurement itself, rather than experimental set‐up (eg coil positioning, localization). This is in contrast to the finding of Lamb et al in their reproducibility study at 1.5 T.^25^ There, they used a 10 cm ^31^P loop for both transmission and reception. In this study we used a larger coil with a rectangular 26 × 28 cm^2^ transmit loop and a flexible 4 × 4 array of 4 cm diameter receive elements. By using a larger coil, we have mitigated some of the challenges associated with placing small loop coils and so our results were less affected.

#### 4.2.3 Per‐subject B_0_ shimming

Per‐subject *B*_0_ shimming improved the precision of the fit of PCr/ATP, as indicated by the lower CRLBs (mean 13.8% for tune‐up versus 10.3% for per subject, *P* = 0.02). However, on our system, the reductions in the linewidths of PCr and γ‐ATP were not significant and there were no improvements in reproducibility (CR 0.67 for tune‐up, 0.66 for per subject). Presumably, uncertainties in optimizing the shim solution counter‐balanced the improved spectral SNR in terms of reproducibility. Additionally, as no cardiac triggering or respiratory gating was used, the calculated shims only apply exactly at one phase in the cardiac and respiratory cycle, which might explain the lack of improvement. It is not therefore immediately obvious whether per‐subject *B*_0_ shimming for cardiac ^31^P‐MRS at 7 T is worth the additional examination time that is required: two iterations of the shimming algorithm adds two approximately 20 s breath holds to the protocol.

If a single 6½ min ^31^P measurement is being included in an examination, then it is likely that per‐subject *B*_0_ shimming is not worth the extra time. In this case, if time permits, better data would probably be obtained by using the time that would have been spent shimming to acquire more averages in the CSI protocol. However, if stress‐response energetics are being monitored (and therefore a CSI protocol is being repeated multiple times while the subject remains in the magnet) then all of the datasets would benefit from performing the per‐subject shimming algorithm at the start of the scan.

Our difficulties with image‐based shimming at 7 T may stem from the inhomogeneous fields produced by the 10 cm surface coil we used. More sophisticated coil designs with better coverage, and dual‐tuned designs, may tip the balance in favour of per‐subject *B*_0_ shimming.^26^

### 4.3 Power and sample size

The reproducibility values presented here enable power and sample size calculations to be performed—an important step in the design of clinical studies. The lower intrasubject variability at 7 T compared with 3 T translates to smaller sample sizes required for sufficient statistical power to detect a given effect. For example, in order to detect a change of 0.2 in PCr/ATP with 80% power in a paired study, power calculations from 3 T data from the Tyler et al^15^ study predict a required sample size of 64, whereas data from this work predicts that only 15 subjects would be required for an equivalent length protocol.

There is some debate in the literature about the possible magnitude of changes in PCr/ATP. Bakermans et al suggested by simulations that the changes in PCr/ATP in healthy subjects upon exercise are on the order of 10%, which they concluded would be impossible to detect by ^31^P‐MRS at 3 T.^24^ Exercise‐induced changes in patients with disease are known to be more substantial, perhaps because their hearts have only a limited energetic reserve because of having to compensate for disease at rest. For example, Betim Paes Leme et al observed a 57% decrease in PCr/ATP in patients with Chagas heart disease on exercise.^27^ Such changes would be clearly detectable at 7 T. It is an open question whether 7 T ^31^P‐MRS can be made sensitive enough to detect changes in high‐energy phosphate energetics in healthy subjects during exercise.

When designing studies, cost is an important factor. Scans at 7 T are more expensive compared with equivalent scans at 3 T: for example, in our centre, the cost of a 7 T scan is about 70% more than a 3 T scan. Despite this, the smaller cohort size required at 7 T for sufficient statistical power would overall lead to a saving in scan fees, eg a 100 × (1–1.70 × 15/64) = 60% saving, using values for the 28 min protocol. Additionally, there would be further savings from the use of fewer consumables and less time spent on patient recruitment. This may also enable a shorter study, delivering more timely information to clinical decision makers. However, at present, our site has more restrictive exclusion criteria for scans at 7 T than at 3 T. Nevertheless, in a recent 7 T ^31^P‐MRS study in patients with DCM^20^ approximately 60% of patients who completed the laboratory screening form were found to have no safety contraindication to MRI at 7 T and were able to participate fully. In our experience, the challenges of patient safety are not insurmountable at 7 T. In particular, we would have excluded far fewer subjects if we had had access to more complete medical device testing data that included 7 T. We expect that this will become available in the coming years.

### 4.4 Limitations

We analysed our results in terms of the PCr/ATP ratio, which is the most commonly measured parameter in cardiac ^31^P‐MRS. However, the PCr/ATP ratio is typically used under the assumption that [PCr] changes while [ATP] remains constant. This assumption is reasonable in the healthy heart, but at high workloads [ATP] decreases and so using the PCr/ATP ratio may obscure changes in ATP concentration.^1^ In future work, methods such as absolute quantitation (ie the concentrations of metabolites are calibrated to recognized units, eg mol/L) would overcome this limitation.^28^

The aim of this work was to assess the reproducibility of cardiac PCr/ATP at 7 T in the healthy population. At 7 T the transmit field strength *B*_1_^+^—and therefore the flip angle achieved in the myocardium—depends strongly on coil loading. Reproducibility may therefore be different in patient groups whose body shape is different from that of the healthy volunteers in this study (eg obese Type 2 diabetes mellitus patients).

## 5 CONCLUSION

We report reproducibility values for human cardiac ^31^P‐MRS at 7 T. These provide the necessary information to design future clinical studies using 7 T ^31^P‐MRS as an endpoint. We evaluated two protocols, one 28 min CSI protocol designed to acquire the best quality spectra in a clinically feasible scan time, and one 6½ min CSI protocol that gives spectra of a quality previously reported at 3 T,^15^ but in a time short enough for use in stress‐response studies. Per‐subject *B*_0_ shimming improved spectral quality, but had a negligible impact on measurement reproducibility.
